# Supplementary material for: Development of Plasmodium falciparum liver-stages in hepatocytes derived from human fetal liver organoid cultures
Source: Nat Commun. 2023 Aug 2;14:4631. doi: 10.1038/s41467-023-40298-7 (PMC10397232; doi:10.1038/s41467-023-40298-7)
Supplement: Supplementary file 1 — Supplementary Information [file 41467_2023_40298_MOESM1_ESM.pdf]

Supplementary Figures

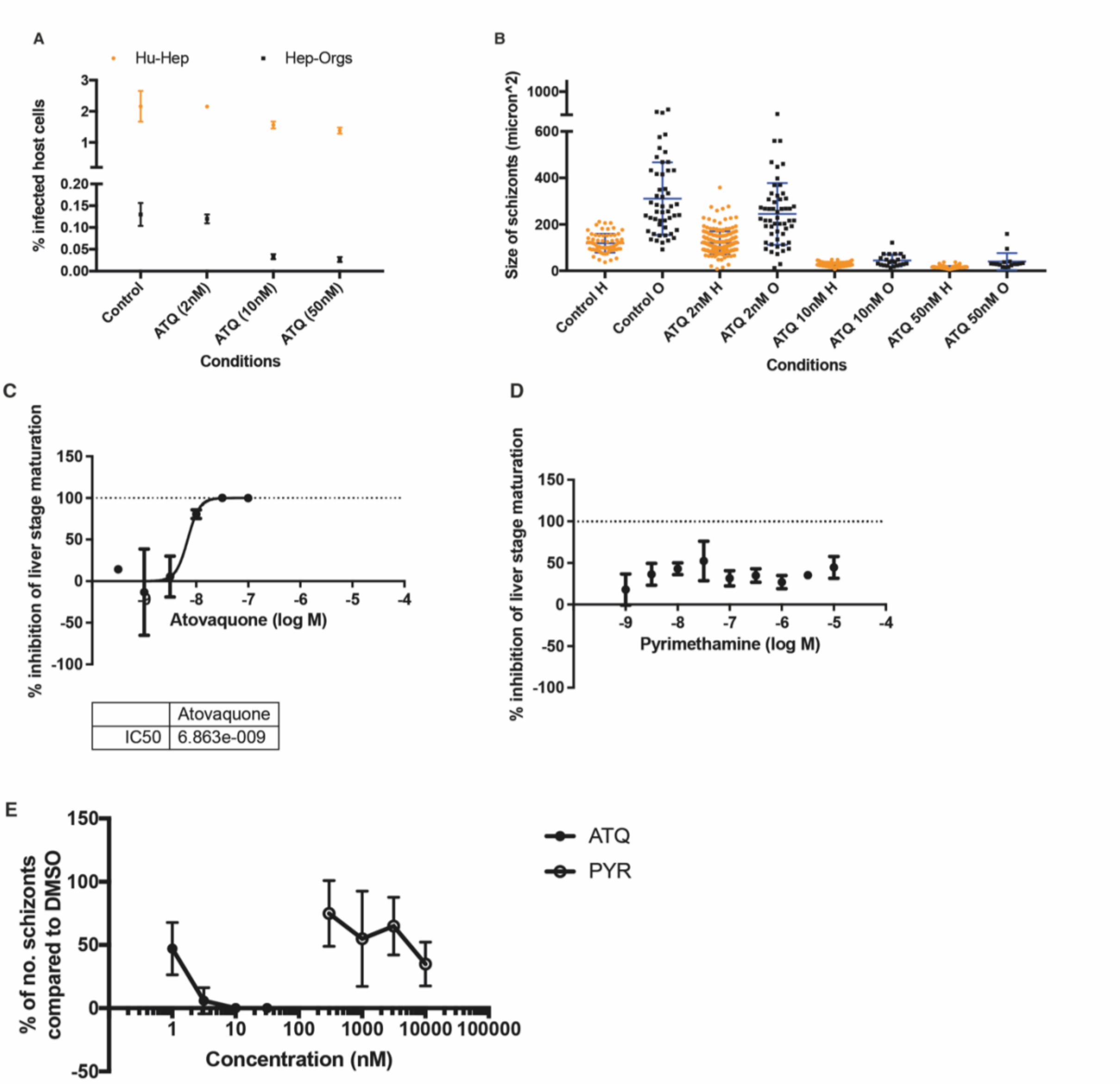

Figure S1: Effect of atovaquone (ATQ) on schizont development in HuHeps and HepOrgs. A) The percentage of infected host cells (HuHep or HepOrg) under different concentrations of ATQ. Each point represents the average of duplicate (HuHep) and triplicate (HepOrg) measurements from one biological replicate (n =1; error bars show the mean ± SD). **B)** The size of the schizonts in HuHep or HepOrg under different concentrations of ATQ. Each point represents the size of a schizont where at least 50 schizonts are measured for each condition: duplicates in HuHeps and triplicates for HepOrgs from one biological replicate (n = 1; Error bar shows the mean ± SD). The H and O denoted after each condition refers to either HuHeps or HepOrgs respectively. Cryopreserved HuHeps infected with PfNF175 and treated with

either **C)** atovaquone or **D)** pyrimethamine at various concentrations (n = 1; error bar shows mean ± SD of three technical replicates). **E)** HepOrgs infected with PfNF175 treated with atovaquone and pyrimethamine (n = 1; error bar shows mean ± SD of three technical replicates).

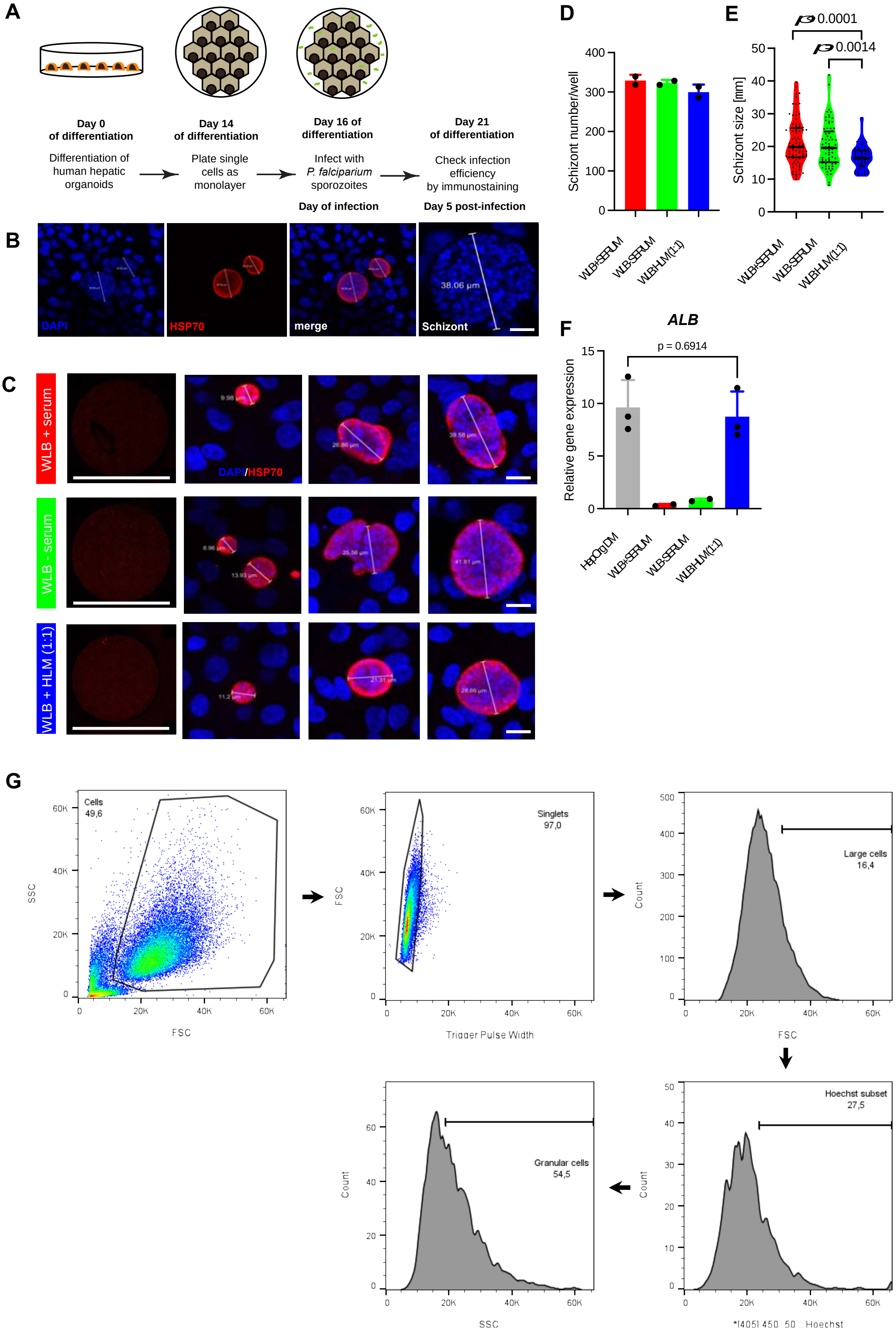

A
Day 0
of differentiation
Differentiation of
human hepatic
organoids
Day 14
of differentiation
Plate single
cells as
monolayer
Day 16 of
differentiation
Infect with
P. falciparium
sporozoites
Day of infection
Day 21
of differentiation
Check infection
efficiency
by immunostaining
Day 5 post-infection
B
DAPI
HSP70
merge
38.06 µm
Schizont
C
WLB + serum
WLB - serum
WLB + HLM (1:1)
DAPI/HSP70
D
Schizont number/well
E
Schizont size [mm]
p 0.0001
p 0.0014
F
ALB
p = 0.6914
Relative gene expression
G
Cells
49,6
SSC
FSC
Singlets
97,0
Trigger Pulse Width
Large cells
16,4
Count
Hoechst subset
27,5
Granular cells
54,5

Figure S2. Media tests and gating strategy for the infection experiments, related to Figure 2. (A) Experimental schematic. (B-D) Immunostaining for schizont marker HSP70 in infected hepatocytes grown in different media (B, C) and the quantification of schizont number (D) and size (E) performed on samples collected day 5 post-infection (*n* = 2 independent experiments). Each dot in D represents one independent experiment. Each dot in E represents an individual measurement (WLB+Serum, n=85; WLB-Serum, n=75; WLB:HLM, n=55). (F) Real-time PCR for A*LB* expression (*n* = 2-3 independent experiments). (G) Gating strategy for the single-cell sorting for RNA-seq. Data are represented as the mean average ± SEM (D), median (Q2), 25th percentile (Q1) and 75th percentile (Q3) (E) and mean average ± SD (F). Two-sided Mann–Whitney U (Wilcoxon rank-sum) test (E) and unpaired two-tailed student's t test (F). Source data are provided as a Source data file. Scale bars in C: left-hand side panels, 5 mm; right-hand side panels, 10 µm.

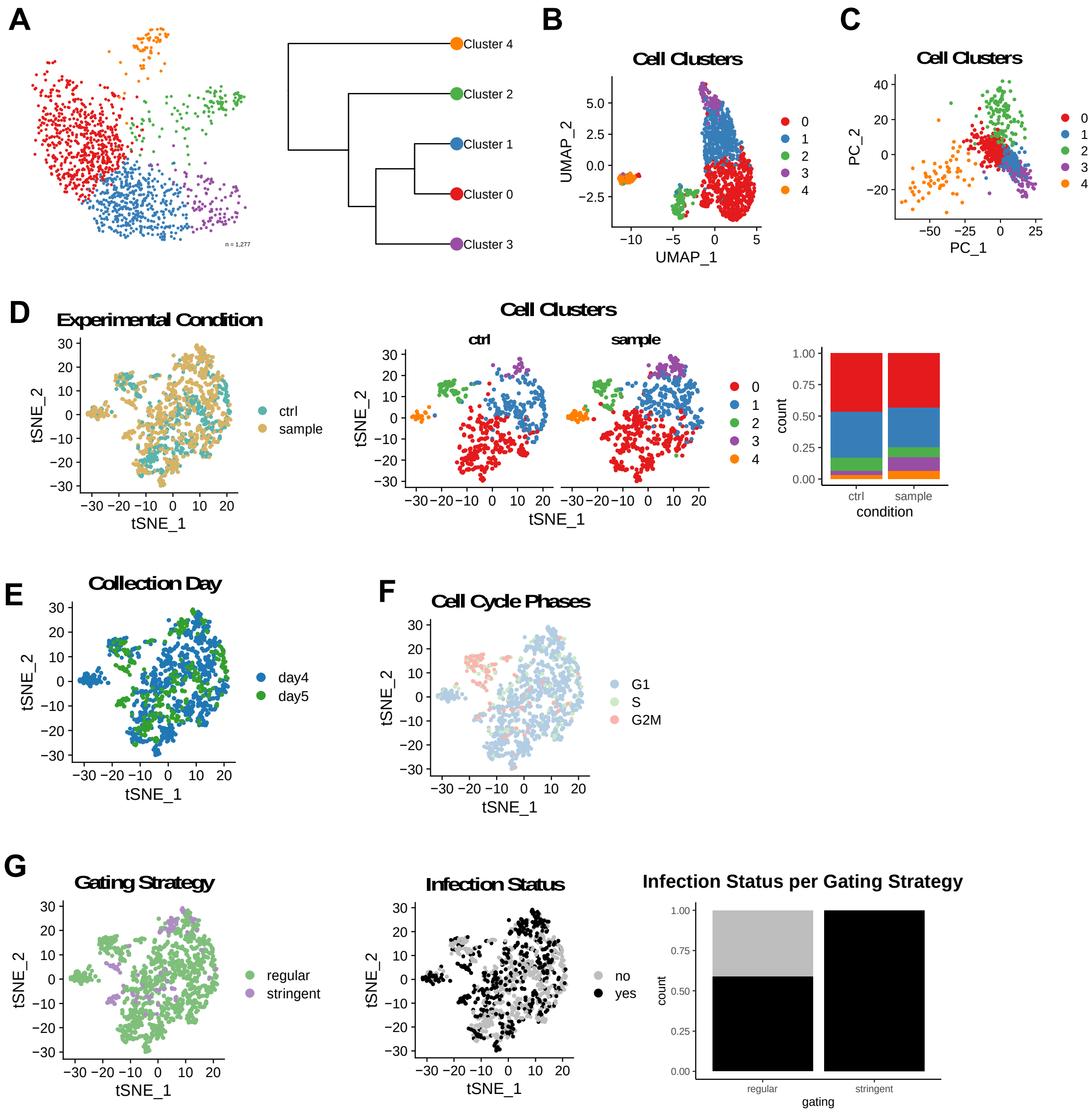

Figure S3. Single-cell transcriptomes of the human reads, related to Figure 2. (A) Shared nearest neighbor (SNN) graph of the 1277 human transcriptomes in the dataset showing the Seurat clusters (left) and cluster tree showing the relationships between the Seurat clusters (right). **(B, C)** UMAP (B) and PCA (C) maps showing the Seurat clusters. **(D)** *t*-SNE maps and cell proportion analysis of experimental conditions (ctrl, n = 533 cells; sample, n = 744 cells) and Seurat clusters. **(E-F)** *t*-SNE maps of the collection day (E; day4, n = 929 cells; day5, n = 348 cells) and cell cycle phases (F; G1, n = 959 cells, S, n = 183 cells; G2M, n = 135 cells). **(G)** Comparison of gating strategy for sample plates end enrichment for infected hepatocyte single cells (regular, n = 1151 cells; stringent, n = 126 cells; no, n = 787 cells; yes, n = 490 cells). Source data are provided as a Source data file and Supplementary Data 1

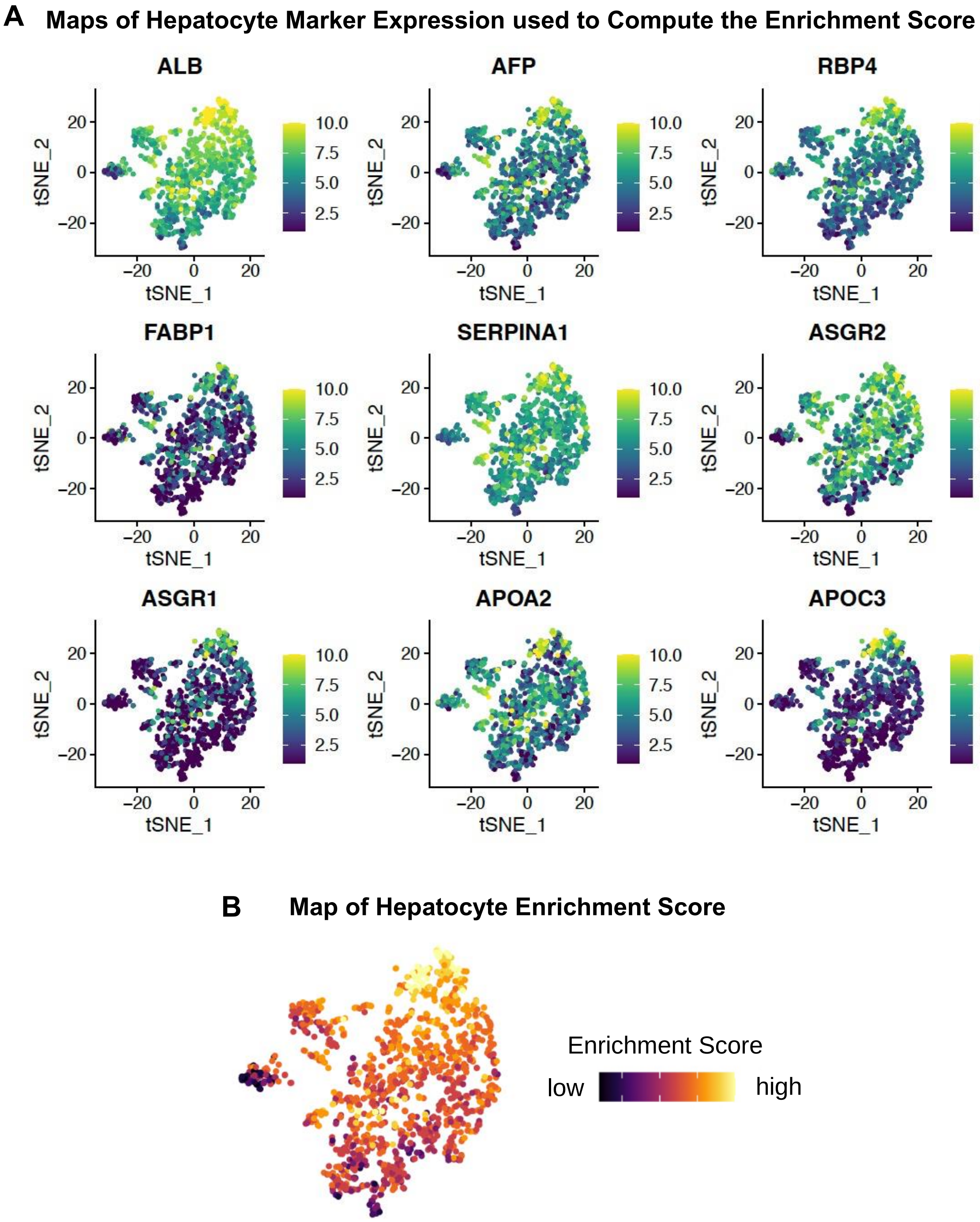

Figure S4. Hepatocyte markers, related to Figure 2. (A, B) *t*-SNE maps (n = 1277 cells) showing the expression of hepatocyte markers (A) as well as the computed hepatocyte enrichment score (B). Source data are provided as a Source data file and Supplementary Data 1.

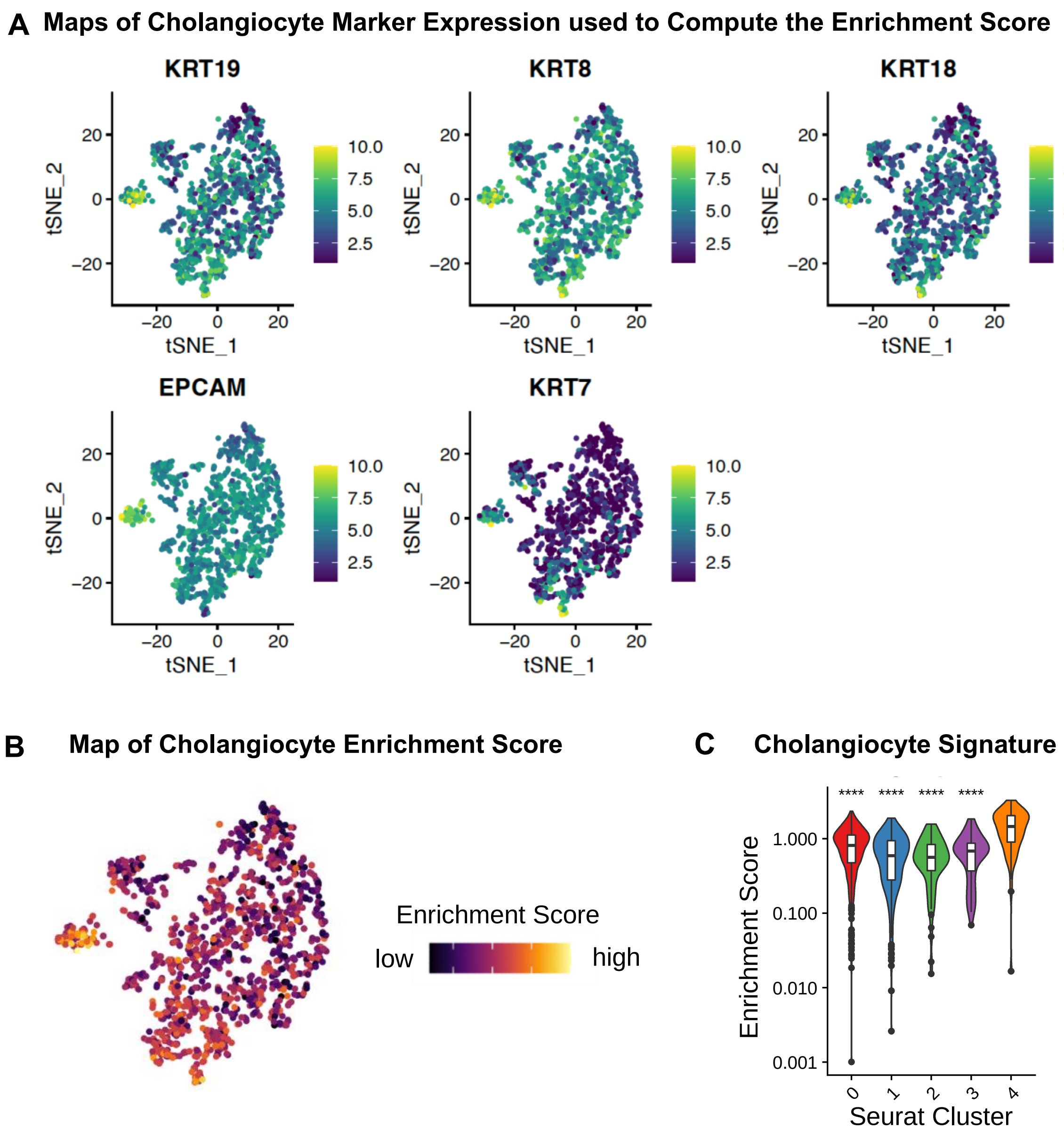

Figure S5. Cholangiocyte markers, related to Figure 2. (A, B) *t*-SNE maps (n = 1277 cells examined) showing the expression of cholangiocyte markers (A) as well as the computed cholangiocyte enrichment score (B). **(C)** Violin plot showing the cholangiocyte enrichment score per Seurat cluster (*n* = 1,277 cells examined). Box plots indicate the median (Q2), 25th percentile (Q1) and 75th percentile (Q3) with the whiskers showing the minimum (Q1 – 1.5 × interquartile range) and maximum (Q3 + 1.5 × interquartile range). Two-sided Mann–Whitney U (Wilcoxon rank-sum) test: **** $p < 0.0001$. Statistics were calculated in comparison to cluster 4: $p < 2.5^{-11}$ (vs cluster 0), $p < 2.4^{-16}$ (vs cluster 1), $p < 1.1^{-12}$ (vs cluster 2), $p < 8.9^{-9}$ (vs cluster 3). Source data are provided as a Source data file and Supplementary Data 1.

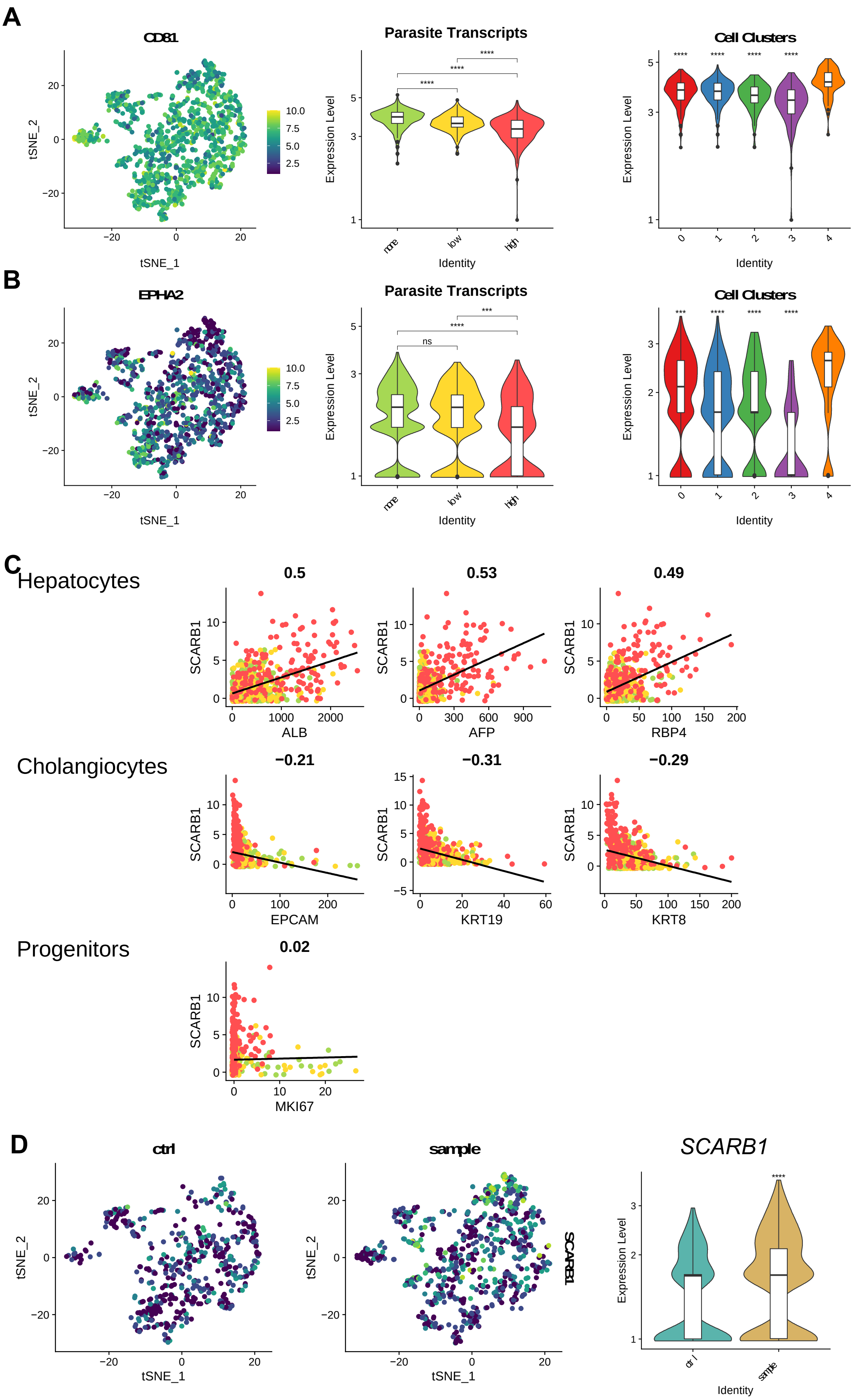
A
CD81
Parasite Transcripts
Cell Clusters
tSNE_2
tSNE_1
Expression Level
Identity
none
low
high
****
ns
***
B
EPHA2
C
Hepatocytes
Cholangiocytes
Progenitors
0.5
0.53
0.49
−0.21
−0.31
−0.29
0.02
SCARB1
ALB
AFP
RBP4
EPCAM
KRT19
KRT8
MKI67
D
ctrl
sample
SCARB1

Figure S6. Entry factors, related to Figure 2. (A, B) *t*-SNE maps and violin plots (n = 1277 cells) showing *the* expression of the genes encoding entry factors CD81 (A) and EPHA2 (B). (C) Correlation of *SCARB1* gene expression against markers of hepatocytes, cholangiocytes and progenitors. (D) t-SNE maps and violin plots showing *SCARB1* expression separated by controls (ctrl) plates (without parasite incubated hepatocytes, n = 533 cells) and sample plates (with parasite incubated hepatocytes, n = 744 cells). Data are represented using *n* = 1,277 cells examined. Box plots indicate the median (Q2), 25th percentile (Q1) and 75th percentile (Q3) with the whiskers showing the minimum (Q1 – 1.5 × interquartile range) and maximum (Q3 + 1.5 × interquartile range). Two-sided Mann–Whitney U (Wilcoxon rank-sum) test: ns $p \geq 0.05$, *** $p < 0.001$, **** $p < 0.0001$. Statistics in (A) and (B) were calculated in comparison to cluster 4. A: $p = 5.1^{-14}$ (none vs low), $p < 2.22^{-16}$ (none vs high), $p = 3.3^{-10}$ (low vs high); $p = 1.2^{-8}$ (vs cluster 0), $p = 8.6^{-9}$ (vs cluster 1), $p < 1.5^{-10}$ (vs cluster 2), $p = 1.8^{-13}$ (vs cluster 3). B: $p = 0.068$ (none vs low), $p = 4^{-9}$ (none vs high), $p = 0.00074$ (low vs high); $p = 0.00087$ (vs cluster 0), $p = 1^{-8}$ (vs cluster 1), $p = 1.7^{-5}$ (vs cluster 2), $p = 1^{-14}$ (vs cluster 3). D: $p = 1.1^{-10}$. Source data are provided as a Source data file and Supplementary Data 1.

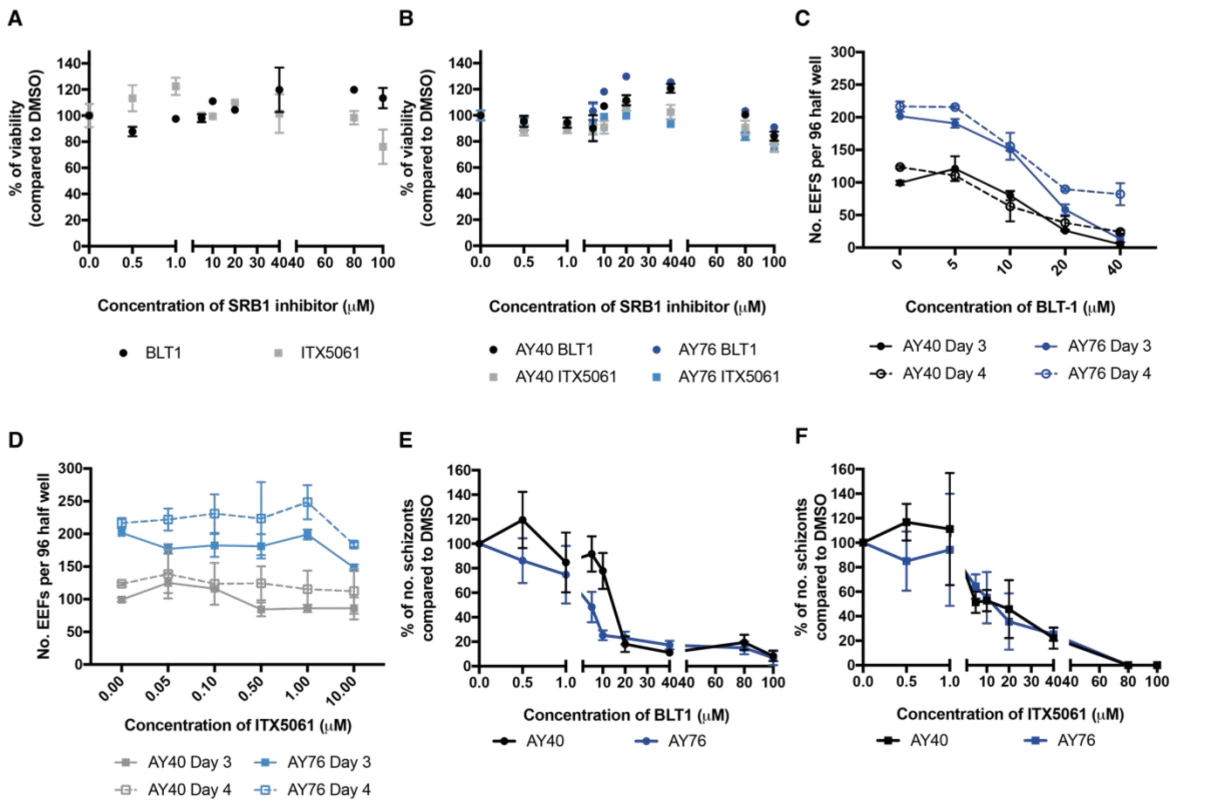

Fig. S7: Dose-dependent effects of SRB1 inhibitors (BLT1 and ITX5061) on cell viability and Pf schizont development. Percentage of viable cells of uninfected differentiated KK2 HepOrg (n =1; error bar is the mean ± SD of three technical replicates) (A) cryopreserved human hepatocytes (n = 2; error bar is the mean ± SD of three technical replicates) AY 40 (black) and AY76 (blue) (B) treated with BLT1 (circle) or ITX5061 (square). Number of PfNF175 schizonts on day 7 p.i. after treatments with either BLT1 (C) or ITX5061 (D) added on either day 3 p.i. (closed) or day 4 p.i. (open) (n = 2; error bar is the mean ± SD of two/three technical replicates). Percentage of Pf schizonts compared to the control (DMSO) in primary cryopreserved hepatocytes AY 40 (blue) or AY 76 (black) treated on day 3 p.i. with either BLT1 (E) or ITX5061 (F). (n = 2; error bar is the mean ± SD of two/three technical replicates)

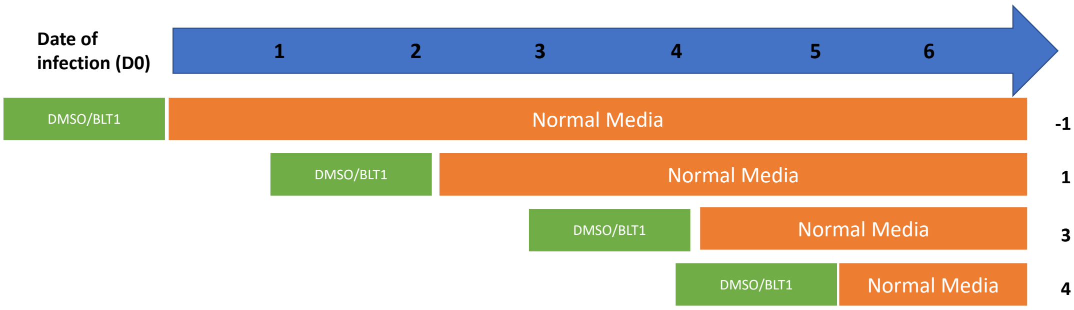

Fig. S8, related to Figure 5. Treatment schedule of BLT1 or DMSO on (un)infected primary hepatocytes. The infection process is stopped on day 7 post invasion.

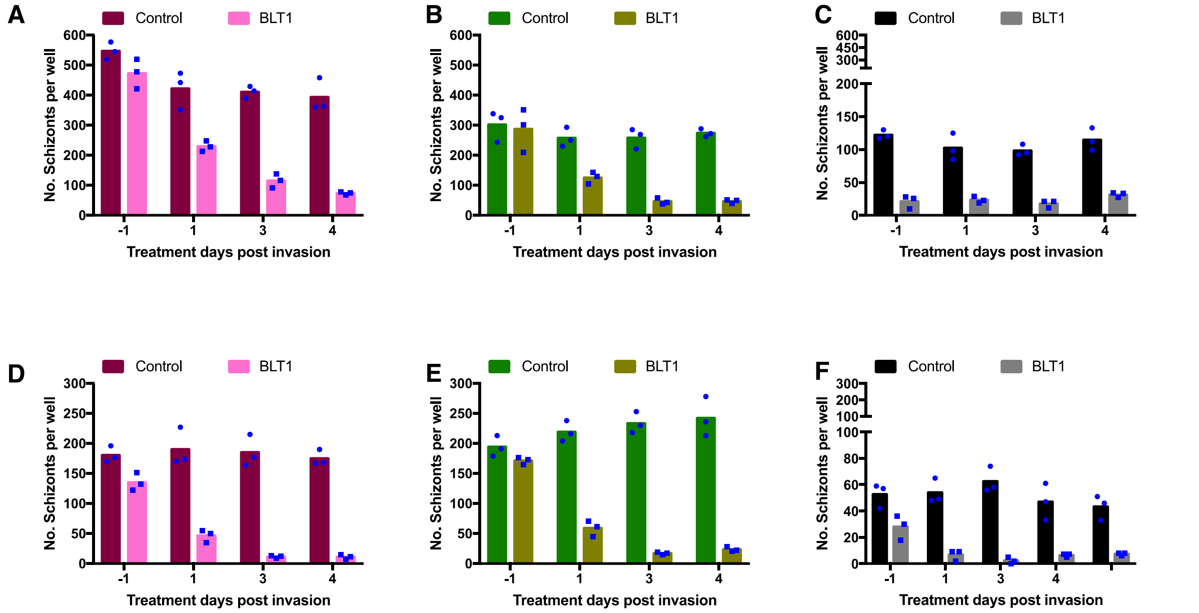

Fig. S9: Effect of BLT1 inhibitor on the absolute number of Pf liver schizonts shown in Figure 5. The number of schizonts in HuHeps infected with NF175 **(A, D)**, NF135 **(B, E)** and NF54 **(C, F)** and treated with either DMSO or BLT1. Each dot represents a technical replicate, and each graph is an independent experiment. In total (per strain), two independent experiments (n = 2) each with 3 technical replicates were performed.

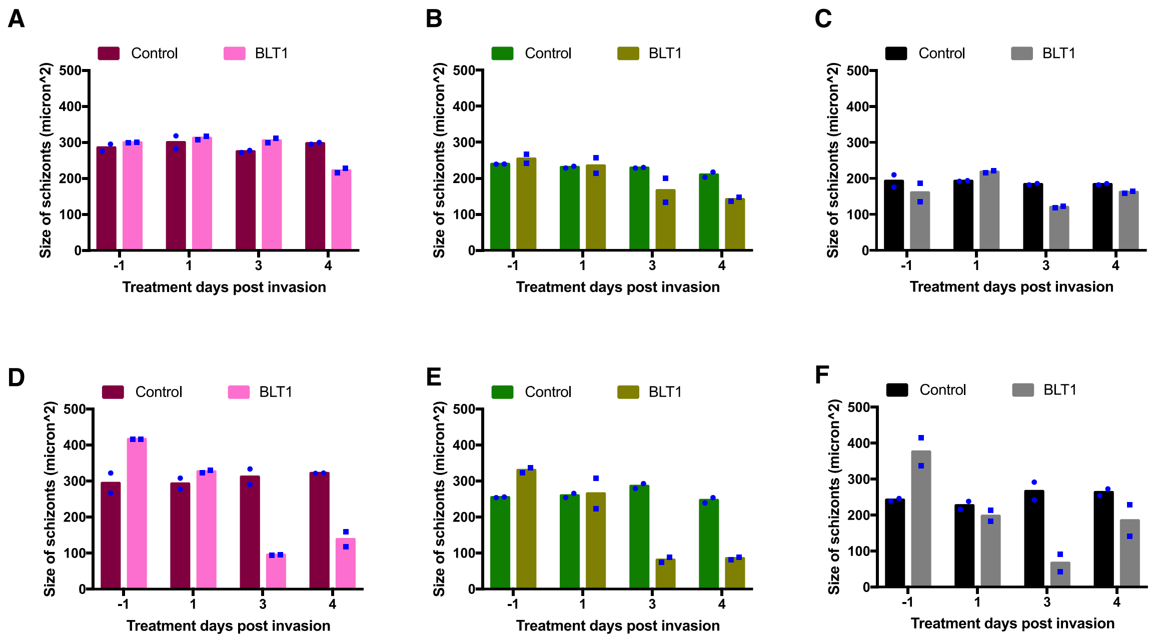

Fig. S10: Effect of BLT1 inhibitor on the sizes of Pf liver stages, related to Figure 5. The size of schizonts in HuHeps infected with NF175 **(A,D)**, NF135 **(B,E)** and NF54 **(C,F)** and treated with either DMSO or BLT1. Each dot represents a technical replicate, and each graph is an independent experiment. In total (per strain), two independent experiments (n = 2) each with 3 technical replicates were performed.

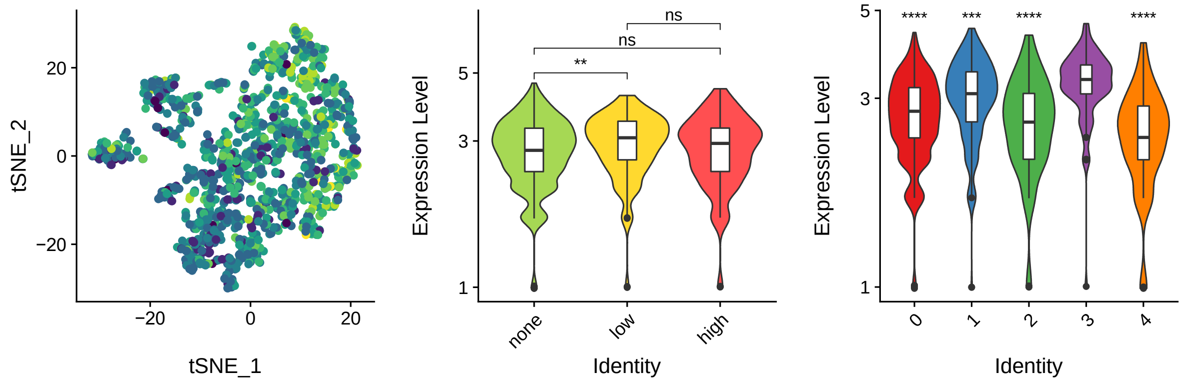

Figure S11. Expression of *GLUL*, related to Figure 2. *t*-SNE maps and violin plots showing *the* expression of the *GLUL* (encoding glutamine synthetase). Data are represented using n = 1,277 cells examined. Box plots indicate the median (Q2), 25th percentile (Q1) and 75th percentile (Q3) with the whiskers showing the minimum (Q1 – 1.5 × interquartile range) and maximum (Q3 + 1.5 × interquartile range). Two-sided Mann–Whitney U (Wilcoxon rank-sum) test: ns $p \geq 0.05$, ** $p < 0.01$, *** $p < 0.001$, **** $p < 0.0001$. Statistics for the right-hand panel were calculated in comparison to cluster 3: p = 0.0011 (none vs low), p = 0.45 (none vs high), p = 0.067 (low vs high); $p < 2^{-16.}$ (vs cluster 0), $p = 5^{-4}$ (vs cluster 1), $p = 6.6^{-13}$ (vs cluster 2), $p = 2.1^{-14}$ (vs cluster 4). Source data are provided as a Source data file and Supplementary Data 1.

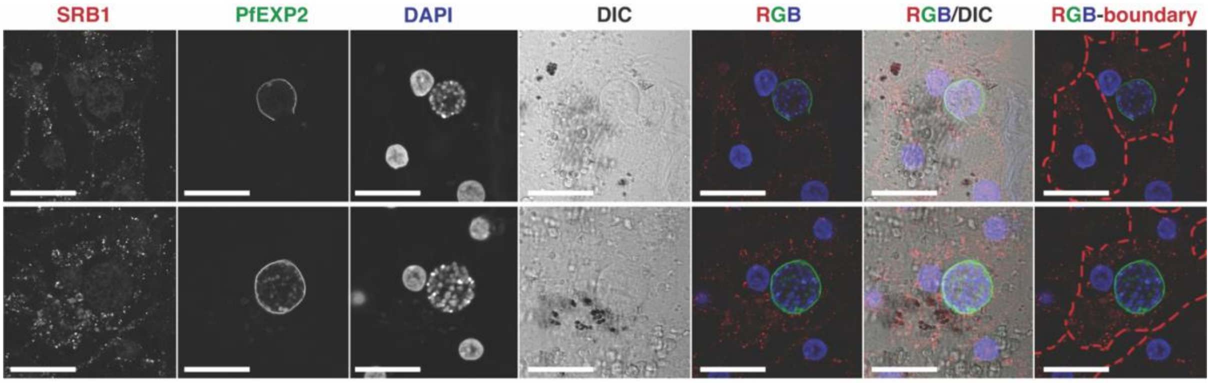

Figure S12: Confocal fluorescence microscopy images of PfNF175 infected primary human hepatocytes on day 5 post infection, stained with PfEXP2 (green: to denote the parasite), human SRB1 (red) and DAPI (blue). Scale bar is 25 microns. These are two representative images from one independent experiment.
