# Supplementary material for: Development of Plasmodium falciparum liver-stages in hepatocytes derived from human fetal liver organoid cultures
Source: Nat Commun. 2023 Aug 2;14:4631. doi: 10.1038/s41467-023-40298-7 (PMC10397232; doi:10.1038/s41467-023-40298-7)
Supplement: Supplementary file 3 — Description of additional supplementary files [file 41467_2023_40298_MOESM3_ESM.docx]

**Description of additional supplementary files**

Supplementary Data 1 – Cluster markers in the dataset of human reads.

Supplementary Data 2 – Differentially expressed genes between infected and uninfected cells in the dataset of human reads.

Supplementary Data 3 – Gene sets upregulated in infected cells in the dataset of human reads.

Supplementary Data 4 – Marker genes of the blood and liver stages in the dataset of Malaria parasite reads.

In all tables, p values (p_val) comparing genes between two groups pof cells were generated using a two-sided Mann–Whitney U (Wilcoxon rank-sum) test. Adjusted p values (p_val_adj) were then generated based on Bonferroni correction using all features in the respective dataset.

Supplementary Data 5 – Meta data to generate plots shown in Figures 2, 3, S3, S4, S5, S6 and S11

Supplementary Data 6 – Meta data of Shared Nearest Neighbor (SNN) coordinates to generate panel Figure S3A

Supplementary Data 7 – Meta data to generate plots shown in Figures 4
